# MAT, a Novel Polyherbal Aphrodisiac Formulation, Enhances Sexual Function and Nrf2/HO-1 Pathway While Reducing Oxidative Damage in Male Rats

**DOI:** 10.1155/2018/8521782

**Published:** 2018-04-29

**Authors:** Kazim Sahin, Mehmet Tuzcu, Cemal Orhan, Hasan Gencoglu, Nurhan Sahin, Fatih Akdemir, Gaffari Turk, Ismet Yilmaz, Vijaya Juturu

**Affiliations:** ^1^Department of Animal Nutrition, Faculty of Veterinary, Firat University, 23119 Elazig, Turkey; ^2^Department of Biology, Faculty of Science, Firat University, 23119 Elazig, Turkey; ^3^Department of Nutrition, Faculty of Fisheries, Inonu University, 44280 Malatya, Turkey; ^4^Department of Reproduction, Faculty of Veterinary, Firat University, 23119 Elazig, Turkey; ^5^Department of Pharmacology, Faculty of Pharmacy, Inonu University, 44100 Malatya, Turkey; ^6^Scientific and Clinical Affairs, OmniActive Health Technologies Inc., Morristown, NJ, USA

## Abstract

*Mucuna pruriens*, Ashwagandha, and* Tribulus terrestris* are known as the enhancers for sexual health, functional activities, vitality, and longevity. These herbs had been widely used in the Ayurveda medicine as aphrodisiacs through the ages, and their efficacy was also verified separately in our previous publication. Therefore, the aim of this study was to determine the effects of* Mucuna*, Ashwagandha, and* Tribulus* complexes on sexual function in rats. Twenty-eight male rats allocated to four groups as follows: (i) negative control (C); (ii) positive control or sildenafil citrate treated group (5 mg/kg) (S); (iii) MAT1 (combination of 10 mg* Mucuna* (M) + 10 mg Ashwagandha (A) + 10 mg* Tribulus* (T)/kg BW); (iv) MAT 2 (20 mg* Mucuna* + 20 mg Ashwagandha + 20 mg* Tribulus*/kg BW). There was no significant difference found between the MAT1 and MAT2 groups while they showed significantly increased testosterone, follicle-stimulating hormone (FSH), and luteinizing hormone (LH) levels when compared to the negative control. Significant increases in Nrf2/HO1 levels and decreases in NF-*κ*B were detected in MAT groups similar to the decrease in serum and testis malondialdehyde (MDA) levels as compared to both controls. The sperm motility, count, and rate also significantly improved in both MAT groups, while ALT, AST, creatinine, ALP, and urea levels did not change in any of the groups. Oral consumption of MATs combination in male rats resulted in inhibition of NF-*κ*B and MDA and also increased sex hormones with Nrf2-mediated HO-1 induction. MAT combinations may improve sexual functions by increasing levels of sexual hormones and regulation of NF-*κ*B and Nrf2/HO-1 signaling pathways.

## 1. Introduction

Infertility and sexual dysfunction are among the most significant health problems encountered by the humans, and almost half of the infertility cases are caused by the male individuals [1, 2]. Oligospermia is a significant problem among the couples that negatively affects the birth rates throughout the world [2, 3]. Normal sexual desire and activity are common for couples until older ages; nevertheless, an increased prevalence of erectile dysfunction and other sexual dysfunctions can often cause serious distress to the aging couples, and seeking a solution to the problem becomes more important [4]. Male sexual dysfunction is generally classified by the etiology of dysfunction, such as vasculogenic, psychogenic, and neurogenic, but, in several cases such as sexual desire disorders, the etiology is complicated [5, 6]. A stressful lifestyle is clearly considered to increase the number of people suffering from one form or another of sexual dysfunction [7]. The use of effective natural antioxidants as supportive treatment has been reported to significantly increase sperm motility and sperm counts in infertile men while reducing oxidative stress [8, 9]. Oxidative stress is the lack of balance between the ability of metabolism to respond or detoxify the detrimental effects of endogenous or exogenous reactive oxygen species (ROS), and, if uncontrolled, a variety of diseases associated with oxidative stress may occur [10, 11]. ROS is the main cause of oxidative stress, which is thought to be an important and reasonable cause of idiopathic male infertility and sexual dysfunction, and oxidative stress-induced sperm DNA damage negatively affects the reproductive outcome [12]. In a previous study where we used* Tribulus*, Ashwagandha, and* Mucuna* separately in rats, we determined that their extracts may be potent enhancers for sexual function and behavior via increasing testosterone levels and regulation of NF-*κ*B and Nrf2/HO-1 pathway with a concomitant decrease in ROS levels as well [13]. Despite the fact that the small amount of ROS is required for healthy sperm functioning, excessive exposure to ROS levels can adversely affect spermatozoa quality and weaken overall fertilization capacity as well [14]. ROS mediated enhanced lipid peroxidation is metabolized to end products malondialdehyde (MDA) and 4-hydroxynonenal (4-HNE) which may cause cellular damage [15]. Transcription factor nuclear factor-kB (NF-*κ*B) is a protein compound which is known as activating in cytokine production and responding to oxidative stress as well as having a major role in apoptosis [16]. It was reported that NF-*κ*B is redox-sensitive and activated by ROS in testicular cells [17]. Nuclear factor erythroid 2-related factor 2 (Nrf2) is a regulator transcription factor which produces a cellular resistance to oxygen-derived free radicals, binds to antioxidant response elements (AREs), responds as a sensor to oxidative and electrophilic stress, and additionally serves in coordination with another antioxidant enzyme such as heme oxygenase-1 (HO-1) [18–20].

Aphrodisiac-effective many plants and their extracts such as* Epimedii herba* (horny goat weed),* Lepidium meyenii *(Maca),* Mucuna pruriens, Panax ginseng, Tribulus terrestris,* and* Withania somnifera *(Ashwagandha) have been reported in the literature to increase sperm number, sexual strength, and testosterone levels while mostly lowering the detriments caused by oxidative stress in the metabolism as well when used at appropriate doses [13, 21]. Thus,* Mucuna pruriens*,* Tribulus terrestris*, and Ashwagandha are aphrodisiac plants used for centuries in traditional Indian and Chinese medicine, which was shown to improve penile erection, develop sperm quality, enhance sexual behaviors, and increase androgen hormone levels in several studies [22–26]. Although there have been many studies on the separate use of* Mucuna pruriens*,* Tribulus terrestris*, and Ashwagandha in the field of male infertility and erectile disorders [13, 22–26], no studies have been found on the combined use of these herbs. In particular, the underlying molecular mechanisms through which combination of these extracts improves above parameters are still unclear. Therefore, the aim of this study was to determine the effects of a novel produced polyherbal formulation of two MAT combinations (two different* Mucuna *+ Ashwagandha +* Tribulus* complexes of 10 mg/kg/BW and 20 mg/kg/BW from each plant species) supplementation on testosterone, reproductive hormones, and male fertility by assessing their effects on sperm characteristics which included the sperm count, motility, viability, and morphology, as well as oxidative stress response and the expressions of testis NF-*κ*B and HO-1/Nrf2 pathways in male rats.

## 2. Materials and Methods

### 2.1. Animals

Twenty-eight male Sprague Dawley rats (*n* = 7, 8 weeks of age, weighing between 180–200 g) obtained from Inonu University, Experimental Animals Lab., Malatya, Turkey. Rats were kept in the cages under normal laboratory environments, including a humidity-measured environment (~50%), along with a 12-hour light/12-hour darkness cycle at an average of 22°C. The rats received ad libitum standard rat diet and water. All the experiments were administered daily from the hours 09.30 am to 17.00 pm for minimizing the effects of environmental alterations. All of the experiments were administered under the Ministry of Health's Guidelines for the Care and Use of Laboratory Animals that was approved by the Inonu University Ethics Committee, Malatya, Turkey.

### 2.2. Experimental Design

After adjusting to the laboratory circumstances, the animals were randomly allocated into the four groups as follows (*n* = 7): (i) Control Group: the rats received standard diet as a negative control; (ii) Sildenafil Group: rats were fed a standard diet with the daily sildenafil citrate administration (5 mg kg/BW; Viagra, Pfizer, USA) via oral gavage as the positive control; (iii) MAT1 Group: the animals were fed with standard diet plus orally given MAT1 combination (10 mg/kg BW* Mucuna* + 10 mg/kg BW Ashwagandha + 10 mg/kg BW* Tribulus*); (iv) MAT2 Group: the animals were fed with standard diet plus orally given MAT2 combination (20 mg/kg BW* Mucuna* + 20 mg/kg BW Ashwagandha + 20 mg/kg BW* Tribulus*). For the MAT1 and MAT2 combinations, dried seed powder of* Mucuna pruriens*, dried fruit extract powder of* Tribulus terrestris*, and dried roots powder of Ashwagandha were provided from the OmniActive Health Technologies (NJ, USA). All the herbals were extracted with 70/30 ethanol-water mixture with a 98% purity. Both MAT combinations were dissolved in distilled water and daily treated to the groups through an intragastric tube in parallel to the positive control group that treated with sildenafil citrate for 8 weeks, which was given on the same basis, based on the published data by us earlier [13].

### 2.3. Sample Collection

At the end of the 8-week study period, blood, epididymis, testis, vas deferens, and ventral prostates samples were collected and weighed. Target organs of the rats were then cleared of any extraneous tissues and placed on a hollow plate with normal saline for washing away the blood. Caudal epididymis was cut longitudinally while being compressed with forceps. Then, the sperm was released by mincing the cauda epididymis into pieces of the Petri dishes containing phosphate-buffered saline (PBS) for sperm analyses. The epididymis was then placed in duplicate, while a cauda epididymis was placed in a Petri dish containing 10 ml of 0.1 M PBS, especially for sperm count and motility analysis, while the other cauda epididymis was placed in another Petri dish containing 1 ml of 0.1 M PBS to determine sperm viability and morphology. The spermatozoa were then allowed to flow out of the cauda epididymis into the buffer. The sperm suspensions were left at room temperature for 10 minutes afterwards so that the sperm swims outside of the cauda epididymis lumen for sperm characteristics analysis.

### 2.4. Sperm Quality

Sperm analysis was done by the methods as previously reported [27]. The sperm count was detected under a light microscope with a hemocytometer. A 10 *μ*l drop of caudal epididymal sperm solution was loaded under a coverslip previously placed on the hemocytometer. For this purpose, another 10 *μ*l caudal epididymal sperm drop was loaded under a new coverslip onto the hemocytometer. Sperm viability was performed on another cauda epididymis sperm samples placed in a Petri dish containing 1 ml of 0.1 M PBS. 3 drops of eosin to 1 drop of sperm suspension slightly stirred together, and one drop of nigrosine was mixed with the solution and a smear acquired after 30 seconds. When the smear dried in the air, ×200 magnification was chosen for the observation. Sperm counts were performed according to the grade of membrane permeability. While the dead sperm heads appeared to be of pinkish coloration, the living sperm was seen a whitish or colorless head.

An equivalent sperm smear, like the one prepared for sperm viability analysis, was also prepared for the sperm morphology examination. To estimate the morphology of the sperm head, neck, and tail, the sperm was detected under ×400 magnification of the imaging microscope. The sperm was characteristically classified by normal or abnormal characterization for the abnormality. The normal sperm was given a score of =100, while the abnormal ones were given =0, to empower the statistical analysis via the Statistical Analysis System (SAS) to be carried out easily.

### 2.5. Laboratory Analyses

Activities of* s*erum alanine aminotransferase (ALT), aspartate aminotransferase (AST), alkaline phosphatase (ALP), and serum urea and creatinine concentrations were examined by a biochemical analyzer (Samsung LABGEO PT10, Samsung Electronics Co, Suwon, Korea). Levels of serum testosterone, follicle-stimulating hormone (FSH), and luteinizing hormone (LH) were analyzed by ELISA analyzer (Elx-800; BioTek Instruments Inc.) with ELISA kits (Cayman Chemical Company, Ann Arbor, Michigan, USA). The concentration of malondialdehyde (MDA) was assessed by High Performance Liquid Chromatography (HPLC; Shimadzu, Kyoto, Japan) equipped with a pump (LC-20 AD), an ultraviolet visible detector (SPD-20A), an inertsil ODS-3 C18 column (250 × 4.6 mm, 5 m), a column oven (CTO-10ASVP), an autosampler (SIL-20A), a degasser unit (DGU-20A5), and a processer system with LC solution Software (Shimadzu).

### 2.6. NF-*κ*B, Nrf-2, and HO-1 Analyses

The western blotting method was used for analyzing the testes NF-*κ*B, Nrf2, and HO-1 levels. For this purpose, testes tissues of the rats precisely were put on the scale and then homogenized in 1 : 10 (w/v) in 10 mM Tris-HCl buffer at pH 7.4, which contained 0.1 mM NaCl, 0.1 mM phenylmethylsulfonyl fluoride, and 5 *μ*M soybean (soluble powder; Sigma, St. Louis, MO, USA) as trypsin inhibitor. The homogenate was then centrifuged at 15,000*g* at 4°C for 30 min, and the supernatant was separated into new tubes. Sodium dodecyl sulfate-polyacrylamide gel electrophoresis (SDS-PAGE) sample buffer containing 2%  *β*-mercaptoethanol was added to the supernatants. After evaluating the equal protein amounts (20 *μ*g) for each sample, the electrophoresis started and subsequently the sample containing gels were transferred to the nitrocellulose membranes (Schleicher and Schuell Inc., Keene, NH, USA). The nitrocellulose membrane blots were washed two times for 5 min in phosphate-buffered saline (PBS) containing 10% of Tween-20 and blocked with 1% bovine serum albumin in PBS for 1 h prior to the application of primary antibody. Rat primer antibodies against NF-*κ*B 65, Nrf-2, and HO-1 were purchased from Abcam (Cambridge, UK). Primary antibodies were diluted (1 : 1000) in the same buffer containing 0.05% Tween-20 (Sigma, St. Louis, MO, USA). The membranes were then incubated overnight at 4°C with the primer antibodies. The blots were washed and incubated with horseradish peroxidase-conjugated goat anti-mouse IgG (Abcam, Cambridge, UK). Protein loading was monitored by a monoclonal mouse antibody against the *β*-actin antibody (A5316; Sigma). Band intensity of protein was calculated using an image analysis system (Image J; National Institute of Health, Bethesda, USA).

### 2.7. Statistical Analysis

Body weight and serum parameters were analyzed by the analysis of variance (ANOVA) to evaluate if any statistical differences existed among groups. If the analysis of variance presented a significant result, Duncan Multiple Range test was done to determine any significant differences between the treatment groups and controls (SAS, 2002). Statistical significance was considered at *P* < 0.05.

## 3. Results

### 3.1. Effects of MAT Combinations on Total Body Weight and Whole and Relative Reproductive Organ Weights

The effects of MAT1 and MAT2 combinations on the alterations of final body weight, total reproductive organ weights, and relative reproductive organ weights are presented (Table 1). There was no significant difference in weight of the testes, whole epididymis, right cauda epididymis, vas deferens, and the final body weights in the groups (*P* > 0.05). The seminal vesicles weight increased in the sildenafil and MAT1 groups than other groups (*P* < 0.01). Relative seminal vesicles weight increased in the MAT1 group by 17% in comparison to the control group (*P* < 0.01). Also, the ventral prostate weight of the sildenafil, MAT1, and MAT2 groups was significantly higher than the control group (*P* < 0.01). However, changes in total and relative weight increases were 38.5% and 41.8% in MAT1 group and 29.4% and 33.3% in MAT2, respectively, according to the control group. In addition, compared to the sildenafil group, the ventral prostate weight increased by 18% and 10% in MAT1 and MAT2 groups, respectively, when (*P* < 0.01) (Table 1).

### 3.2. Effects of MAT Combinations on Sperm Characteristics and Abnormal Sperm Rate

The effects of MAT1 and MAT2 combinations on sperm characteristics and abnormal sperm rate percentage are presented (Table 2). The effects of MAT1 and MAT2 on sperm motility were not statistically different from sildenafil group but were significantly higher than the control group (*P* < 0.0001). In addition, compared to the control group, sperm motility increased by 29.5% and 30.4% in MAT1 and MAT2 groups (*P* < 0.001). However, the sperm counts increased by 29.8% in MAT1 and by 48.1% in MAT2 groups when compared to the control group. These increases were 1.30-fold in MAT1 and 1.48-fold in MAT2 compared to the control group (*P* < 0.01). There was no statistical difference in the head and total anomaly ratios between the groups (*P* > 0.05), whereas tail anomalies were notable in the MAT2 group compared to the MAT1 group but not in the control and sildenafil groups (*P* < 0.05), (Table 2).

### 3.3. Effects of MAT Combinations on Liver Enzymes, Urea, and Creatinine

The effects of MAT1 and MAT2 combinations on the liver function test enzymes aspartate aminotransferase (AST), alanine aminotransferase (ALT), and alkaline phosphatase (ALP), along with the kidney function test markers blood urea nitrogen (BUN) and creatinine are presented (Table 3). There was not any statistical difference between the groups regarding all these markers (*P* > 0.05) (Table 3).

### 3.4. Effects of MAT Combinations on FSH, LH, and Testosterone

The effects of MAT1 and MAT2 combinations on the levels of follicle-stimulating hormone (FSH), luteinizing hormone (LH), and testosterone levels are shown (Table 4). Compared with the control group, FSH levels were higher in MAT1, MAT2, and sildenafil groups, but there was no difference between MAT1, MAT2, and sildenafil groups (*P* < 0.0001). The highest LH levels were found in MAT2 and sildenafil groups (*P* < 0.01). However, there was no statistically significant difference in LH levels between these groups and MAT1 (*P* > 0.05). Besides, the MAT1 group showed a 50% luteinizing hormone increase compared to the control group, while MAT2 showed 72.7%. While the sildenafil-treated group showed the highest testosterone levels, both the MAT1 and MAT2 groups showed more significant testosterone levels increase than the control group (*P* < 0.0001). The increase in testosterone levels compared to the control group was 1.52-fold for MAT1 and 1.70-fold for MAT2, (Table 4).

### 3.5. Effects of MAT Combinations on Serum and Testis MDA Levels

The effects of MAT1 and MAT2 combinations on lipid peroxidation end product malondialdehyde (MDA), in the sera and testes, are presented (Table 4). The lowest serum MDA levels were found in the MAT1 and MAT2 groups, and the highest MDA levels were in the sildenafil and control groups (*P* < 0.0001). Compared to the control and sildenafil group, the decrease in serum MDA levels in the MAT1 group was 27% and 28%, respectively, and, in the MAT2 group, this decrease was 35% and 36%, respectively, (Table 4).

Similar to serum results, it was found that the highest levels of testicular tissue MDA were found in sildenafil and control groups, the MAT1 group was significantly lower than these groups, and the MAT2 group had the lowest MDA level among all groups (*P* < 0.0001). This reduction in MDA levels was 13.8% and 16.2% for MAT1 and was 19 and 21.2% for MAT2 compared to control and sildenafil groups, respectively, (Table 4).

### 3.6. Effects of MAT Combinations on NF-*κ*B, Nrf-2, and HO-1 Levels

**Figure 1:**
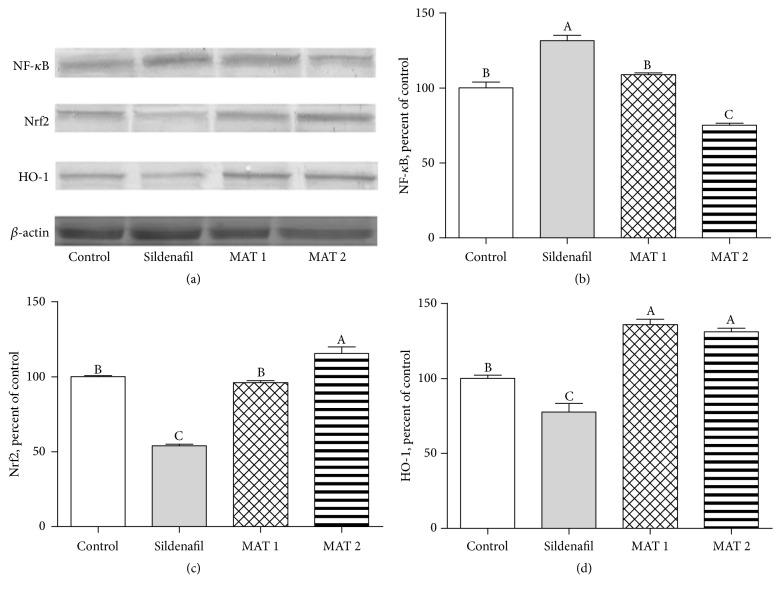
Western blot bands of NF-*κ*B, Nrf-2, HO-1, and *β*-actin (Panel (a)), together with NF-*κ*B (Panel (b)), Nrf-2 (Panel (c)), and HO-1 (Panel (d)) comparative levels among the groups in male rats (*P* < 0.0001). Data are expressed as a ratio of normal control value (set to 100%). Blots were repeated at least 4 times (*n* = 4) and a representative blot is shown. *β*-Actin band is included as a housekeeping protein to confirm equal protein loading. The bars describe the mean and standard error. Data points with different superscripts significantly differ at the level of *P* < 0.05 by one-way ANOVA and post hoc Tukey test.

The effects of MAT1 and MAT2 combinations on NF-*κ*B, Nrf-2, and HO-1 protein levels are shown in Figure 1. NF-*κ*B levels were found to be the highest at sildenafil group while being found significantly lower than sildenafil at both control and MAT1 groups, subsequently the least at MAT2 group (*P* < 0.0001). The significant decrease of NF-*κ*B levels in MAT2 group was found to be 24.9% more when compared to the control group and also 42.9% more when compared to the sildenafil group (Figure 1).

Nrf2 levels were not different between the control group and MAT1 groups; however, in the sildenafil group, there was a significant increase in Nrf2 levels compared to these groups. The highest Nrf2 level was found in the MAT2 group (*P* < 0.0001). Compared to the sildenafil group, Nrf2 levels increased by 1.78- and 2.14-fold in MAT1 and MAT2 groups. The highest levels of HO-1 were found in the MAT1 and MAT2 groups, while the lowest HO-1 levels were found in the sildenafil group (*P* < 0.0001). Significant increases in the MAT1 and MAT2 groups were 1.36- and 1.31-fold higher than the control group and 1.75- and 1.69-fold higher than the sildenafil group, respectively (Figure 1).

## 4. Discussion

Studies conducted with* Mucuna pruriens*,* Withania somnifera* (Ashwagandha), and* Tribulus terrestris* have provided reliable evidence on improving sexual dysfunction and sperm abnormalities for decades [22, 24–28]. The results of the present study have showed that the combined effects of these three different sexual aphrodisiac plants (MAT), as we previously demonstrated in another study to be very effective separately [13], also have been proved to be very successful and somewhat particularly better effective in increasing sexual capacity by remarkably enhancing testosterone levels and antioxidant protection efficacy via promoting the Nrf-2 and HO-1 levels while lowering lipid peroxidation and NF-*κ*B levels as well (Figure 1). In our previous study, these three plant supplements were successful in similar proportions and although we have found* Tribulus *extract to be relatively better, it is inevitable to take advantage of certain nutritional supplements as a mixture, which is easier due to individually difficulties to obtain and benefit from.

In the current study, no differences were observed in the testes, whole epididymis, right cauda epididymis, vas deferens, and final body weights between both absolute and relative weights of all the groups, whereas the seminal vesicles and ventral prostate weight changes showed significance (Table 1). Seminal vesicle and prostate are the androgenic glands of the male genital tract and both of them produce the majority of the seminal secretions [29]. Especially in the MAT2 group, the mean seminal vesicle weight was nearly at the control group levels while MAT1 showed no difference compared to positive control group sildenafil. Similar to our MAT2 results, in an earlier study, seminal vesicle occlusion of male mice resulted in an increased sexual activity of the animals [30]. Although not significant in seminal vesicles, both absolute and relative weight gain in the ventral prostates of the MAT groups were found not different compared to the sildenafil group in the study term, which may be related because of the increased semen production of treated animals [31, 32]. Except for this situation, as an addition to sexual dysfunction and low sperm production in men, prostate weight gain is generally due to a variety of adverse factors in long term such as age-related or high-fat diets and special diet content factors are able to affect the development of these type of diseases [33–35].

In the present study, it was determined that sperm count and motility of both MAT treated groups were significantly increased statistically at the same level when compared to the control group (Table 2). In accordance with these results, it has been reported that the mammalian metabolism can produce more active and much more sperm in the testes by the aid of various antioxidants such as vitamins A, E or potential natural antioxidant sourced fruits and their active components [36–38]. In addition, the organic compound of S-allylcysteine (SAC), a natural component of fresh garlic, has been assessed in the male rats against the oxidative stress and aging-related sperm dysfunction and was found to substantially increase the number and quality of spermatozoa as well [39].

In this study, no difference was found between the groups in the serum levels of liver and kidney damage markers alkaline phosphatase (ALP), alanine aminotransferase (ALT), aspartate aminotransferase (AST), blood urea nitrogen (BUN), and creatinine (CRE) (Table 3). The levels of ALP, ALT, and AST in the serum are clinically used as biochemical markers to elucidate the liver damage [40, 41]. Furthermore, BUN and CRE are endogenous byproducts that are released into the body fluids and discarded by glomerular filtration, and their levels are clinically used to express the physical condition of the kidneys [42, 43]. Our results on these levels have been found to be consistent with several similar studies along with a parallel study on the ethnopharmacological efficacy of a different polyherbal mixture which was also shown to be protective in the liver and kidneys even against acute acetaminophen (APAP) damage when used at appropriate doses [44–46].

The generation of male gametes is directly linked with the collective actions of both follicle-stimulating hormone (FSH) and luteinizing hormone (LH), the two separate heterodimeric glycoprotein gonadotropin hormones on the testis [47]. While FSH is expressed in the Sertoli cells, the action of LH is provided via the production of testosterone by the Leydig cells [48]. In the present study, FSH and LH levels of the male rats were significantly increased in MAT1 and MAT2, when compared to the control group. However, the testosterone levels were also significantly increased in both groups given the MAT combinations, compared to the control group, following the sildenafil group (Table 4). Similar to our results, in a study with methanolic extract of* Tribulus terrestris* fruit alone against testicular toxicity, it was found that oral pretreatment of the extract was protective and antioxidant effective against intoxication and that effect against testicular toxicity was thought to have occurred because of the increased release of testosterone, FSH, and LH as well as an increase in tissue antioxidant capacity [49]. It was also shown that oral consumption of* Tribulus terrestris* could significantly reverse the reduction of sex hormones and gonadotropins in the male rats due to the morphine addiction [50]. Similar results have been obtained in some recent studies of sexual functioning with different antioxidant-acting compounds, in terms of FSH, LH, and testosterone levels [51, 52].

Malondialdehyde (MDA) is being used for a long time as a biomarker for the lipid peroxidation of both omega-3 and omega-6 fatty acids due to its easy reaction with thiobarbituric acid (TBA) and widely accepted as a marker for oxidative stress [53]. In the present study, both MAT combinations significantly decreased in terms of MDA levels compared to control (Table 4). In particular, MAT2 showed a more effective decline in testicular tissue MDA levels than the all groups. Consistently,* Mucuna pruriens*, Ashwagandha, and* Tribulus terrestris* separately reduced MDA levels in many studies with or without an aphrodisiac property or a disease model, thanks to their effective antioxidant properties [13, 54–56].

Heme oxygenase-1 (HO-1) is a substantial member of phase II detoxification enzymes which detoxify overreactive radicals and therefore protect from excessive oxidative stress and the nuclear factor erythroid 2-related factor 2 (Nrf2) is known to be a major component in the induction of HO-1 [57]. Nrf2 and NF-*κ*B are suggested as the key molecules which modulate the cellular redox condition and the adjustment of inflammation and stress responses [58]. The interaction among these cellular pathways takes place via a series of complicated molecular interplay and is usually dependent on the tissue context and type of cells, such as NF*κ*B's ability to use histone deacetylase 3 (HDAC3), which blocks Nrf2 signaling, thus leading to local hypoacetylation [58, 59]. In the present study, testis tissue western blot results showed significant promotions in the levels of Nrf-2 and HO-1 and reductions in the NF-*κ*B levels of the MAT2 group followed by MAT1 in comparison to the control group (Figure 1). In a previous study by our group, it was found that separately oral treatment of* Mucuna pruriens*, Ashwagandha, and* Tribulus terrestris* could increase the levels of Nrf2/HO-1 and reduce NF-*κ*B in the reproductive organ parts as well [13]. In a recent study, in which a different antioxidant effective plant formulation was combined, the Nrf2/HO-1 pathway was upregulated while NF-*κ*B was downregulated against the oxidative stress in the rat tissues similarly to our study, and the effect of the triple plant combination was found to be better than the effect of each one or a double combination as well [60].

In conclusion, the present study shows that the combinations of MAT and especially MAT2, exerted significant improving effects on sexual performance capacity, and these effects are mostly via the increment of the antioxidant status and the modulation of NF-*κ*B and Nrf2 signaling pathways. The future studies required to assess the effectiveness and safety of these medicinal plants, separately or in combination, to achieve more precise results and dose evaluation.

## Figures and Tables

**Table 1:** The effects of extracts on final body weight and absolute and relative reproductive organ weights.

Item	Groups			
	Control	Sildenafil	MAT I	MAT II
Final body weight, g	267.00 ± 4.57	254.67 ± 10.26	262.50 ± 12.65	260.83 ± 9.44
Testis, g	1.178 ± 0.017	1.174 ± 0.029	1.227 ± 0.068	1.257 ± 0.058
Whole epididymis, g	0.473 ± 0.007	0.468 ± 0.020	0.462 ± 0.018	0.418 ± 0.015
Right cauda epididymis, g	0.170 ± 0.003	0.172 ± 0.005	0.185 ± 0.014	0.175 ± 0.010
Vas deferens, g	0.109 ± 0.004	0.112 ± 0.008	0.118 ± 0.010	0.117 ± 0.012
Seminal vesicles, g	1.313 ± 0.094^ab^	1.575 ± 0.070^a^	1.508 ± 0.089^ab^	1.203 ± 0.050^b^
Ventral prostate, g	0.408 ± 0.027^b^	0.478 ± 0.037^ab^	0.565 ± 0.027^a^	0.528 ± 0.026^a^
Testis^*∗*^, %	0.442 ± 0.011	0.460 ± 0.022	0.469 ± 0.022	0.483 ± 0.020
Whole epididymis^*∗*^, %	0.178 ± 0.003	0.184 ± 0.012	0.178 ± 0.009	0.185 ± 0.007
Right Cauda epididymis^*∗*^, %	0.064 ± 0.001	0.067 ± 0.004	0.071 ± 0.004	0.067 ± 0.003
Vas deferens^*∗*^, %	0.041 ± 0.002	0.044 ± 0.003	0.045 ± 0.003	0.045 ± 0.004
Seminal vesicles^*∗*^, %	0.493 ± 0.037^bc^	0.621 ± 0.026^a^	0.577 ± 0.030^ab^	0.462 ± 0.013^c^
Ventral prostate^*∗*^, %	0.153 ± 0.009^b^	0.187 ± 0.010^ab^	0.217 ± 0.011^a^	0.204 ± 0.014^a^

^*∗*^Relative reproductive organ weights [organ weight (g)/final body weight (g) × 100]; data are means ± SE. Different superscripts (a–c) indicate group mean differences (*P* < 0.05).

**Table 2:** The effects of extracts on sperm characteristics and abnormal sperm rate, %.

Item	Groups			
	Control	Sildenafil	MAT I	MAT II
Motility, %	63.88 ± 2.00^b^	73.98 ± 4.13^ab^	82.75 ± 3.59^a^	83.33 ± 2.11^a^
Count^*∗*^	111.67 ± 2.65^b^	152.80 ± 11.22^a^	145.00 ± 12.77^ab^	165.33 ± 5.90^a^
*Abnormal sperm rate, %*				
Head	6.83 ± 1.52	6.40 ± 2.54	5.83 ± 0.87	5.00 ± 1.37
Tail	4.67 ± 1.02^ab^	4.60 ± 0.93^ab^	3.17 ± 1.08^b^	8.50 ± 1.80^a^
Total	11.50 ± 1.80	11.00 ± 3.13	9.00 ± 1.75	13.50 ± 1.15

Data are means ± SE. Different superscripts (a, b) indicate group mean differences (*P* < 0.05); ^*∗*^million/right cauda epididymis.

**Table 3:** The effects of extracts on serum biochemical parameters.

Item	Groups			
	Control	Sildenafil	MAT I	MAT II
ALP, U/L	193.71 ± 4.67	191.57 ± 3.76	203.29 ± 4.19	204.57 ± 3.88
ALT, U/L	45.43 ± 2.06	47.57 ± 2.97	44.57 ± 2.36	44.00 ± 2.43
AST, U/L	93.43 ± 3.34	100.71 ± 3.78	93.29 ± 3.38	92.00 ± 2.88
BUN, mg/dl	33.57 ± 0.75	33.86 ± 0.63	33.14 ± 0.80	33.29 ± 0.57
Creatinine, mg/dl	0.30 ± 0.01	0.32 ± 0.01	0.30 ± 0.01	0.29 ± 0.01

ALP: alkaline phosphatase; ALT: alanine aminotransferase; AST: aspartate aminotransferase; BUN: blood urea nitrogen; data are means ± SE.

**Table 4:** The effects of extracts on serum gonadotropic/androgenic hormones and MDA levels.

Item	Groups			
	Control	Sildenafil	MAT I	MAT II
FSH, mIU/ml	0.28 ± 0.03^b^	0.45 ± 0.03^a^	0.38 ± 0.02^a^	0.44 ± 0.02^a^
LH, mIU/ml	0.22 ± 0.05^b^	0.40 ± 0.04^a^	0.33 ± 0.02^ab^	0.38 ± 0.02^a^
Testosterone, ng/ml	2.27 ± 0.08^c^	4.35 ± 0.17^a^	3.46 ± 0.05^b^	3.85 ± 0.07^b^
Serum MDA, *µ*mol/L	0.60 ± 0.02^a^	0.61 ± 0.02^a^	0.44 ± 0.02^b^	0.39 ± 0.02^b^
Testis MDA, nmol/g	1.74 ± 0.02^a^	1.79 ± 0.03^a^	1.50 ± 0.01^b^	1.41 ± 0.01^c^

FSH: follicle-stimulating hormone; LH: luteinizing hormone; MDA: malondialdehyde. Data are means ± SE. Different superscripts (a–c) indicate group mean differences (*P* < 0.05).
